# Clinical and regulatory development strategies for *Shigella* vaccines intended for children younger than 5 years in low-income and middle-income countries

**DOI:** 10.1016/S2214-109X(23)00421-7

**Published:** 2023-10-17

**Authors:** Birgitte K Giersing, Richard Isbrucker, David C Kaslow, Marco Cavaleri, Norman Baylor, Diadié Maiga, Patricia B Pavlinac, Mark S Riddle, Gagandeep Kang, Calman A MacLennan

**Affiliations:** a Department of Immunization, Vaccines and Biologicals, World Health Organization, Geneva, Switzerland; b Norms and Standards for Biologicals, World Health Organization, Geneva, Switzerland; c Essential Medicines and PATH Center for Vaccines Innovation and Access, PATH, Seattle, WA, USA; d Office of Health Threats and Vaccine Strategy, European Medicines Agency, Amsterdam, Netherlands; e Biologics Consulting Group, Alexandria, VA, USA; f Vaccine Regulation, World Health Organization, Regional Office for Africa, Brazzaville, Republic of the Congo; g Global Center for Integrated Health of Women, Adolescents, and Children (Global WACh), Department of Global Health and Department of Epidemiology, University of Washington, Seattle, WA, USA; h Department of Internal Medicine (Community Faculty), University of Nevada, Reno, NV, USA; i Department of Gastrointestinal Sciences, CMC Vellore, Vellore, India; j Enterics, Diagnostics, Genomics & Epidemiology, Global Health, Bill & Melinda Gates Foundation, Seattle, WA, USA

## Abstract

Shigellosis causes considerable public health burden, leading to excess deaths as well as acute and chronic consequences, particularly among children living in low-income and middle-income countries (LMICs). Several *Shigella* vaccine candidates are advancing in clinical trials and offer promise. Although multiple target populations might benefit from a *Shigella* vaccine, the primary strategic goal of WHO is to accelerate the development and accessibility of safe, effective, and affordable *Shigella* vaccines that reduce mortality and morbidity in children younger than 5 years living in LMICs. WHO consulted with regulators and policy makers at national, regional, and global levels to evaluate pathways that could accelerate regulatory approval in this priority population. Special consideration was given to surrogate efficacy biomarkers, the role of controlled human infection models, and the establishment of correlates of protection. A field efficacy study in children younger than 5 years in LMICs is needed to ensure introduction in this priority population.

## Introduction

*Shigella* remains the leading bacterial cause of diarrhoea, accounting for approximately 10% of the 1·5 million estimated diarrhoea deaths annually.^1^ *Shigella* infections occur worldwide, with broad geographical distribution and across all age groups. However, the greatest burden of morbidity and mortality is among children younger than 5 years in low-income and middle-income countries (LMICs); in 2019, *Shigella* infections were responsible for an estimated 94 000 deaths,^1^ with peak incidence in the second year of life. For young children, both symptomatic and asymptomatic infections can induce or exacerbate growth stunting, resulting in long-term adverse consequences on physical and cognitive development, and increasing risk of mortality from other infectious diseases.

In addition to its impact on children, *Shigella* can cause severe illness among travellers, deployed military personnel, and expatriates in LMICs and is associated with reactive arthritis and irritable bowel disease.^2^ *Shigella* infections also have the potential to cause large outbreaks in both younger and older age groups,^3^ and are important sexually transmitted infections.^4^ The rise of antibiotic-resistant enteric bacteria, as well as the overuse of empiric antibiotics for viral enteric infections, has made the need for effective vaccines against *Shigella* an even greater public health priority. This broad burden represents a potentially large market for *Shigella* vaccines, and is a key factor in investment by manufacturers. However, WHO is primarily focused on defining the pathway and evidence needs for the development of safe, effective, and affordable vaccines to reduce mortality and morbidity due to dysentery and diarrhoea caused by *Shigella* in children younger than 5 years in LMICs.^5^ This strategy includes determination of the most expeditious route to regulatory approval for this target population, but also aims to ensure that the evidence is available to position these vaccines for efficient policy recommendation, and timely implementation. As such, WHO has developed preferred product characteristics for *Shigella* vaccines that describe the desired attributes for vaccines to be used in LMIC contexts.^5^

Several vaccine candidates are currently in, or approaching, phase 2 clinical proof-of-concept studies,^6^ and the potential subsequent clinical trial designs and pathways to regulatory approval have become a crucial question for vaccine developers and funders. The controlled human infection model (CHIM) for *Shigella* has had and might continue to have an important role as a de-risking strategy for early candidate advancement, serving as a tool to compare candidates, expand our understanding of pathogen–host responses and inform the establishment of correlates of protection. However, *Shigella* CHIMs are limited to use in adults. Although they might have a pivotal role in accelerating the regulatory approval in adult populations in high-income countries (HICs) exposed to high-risk settings, they are considered to have limited applicability in informing decision making for introduction of first-in-class *Shigella* vaccines in infants and young children in LMICs. Nevertheless, earlier regulatory approval of adult vaccines in HICs, potentially based on CHIMs, enhances the investment case for manufacturers and presents an opportunity to build acceptance and create demand for this new class of vaccines, including for use in infants and children.

To explore the potential regulatory approval and introduction pathways for *Shigella* vaccines, and specifically the role of the *Shigella* CHIM in both regulatory approval and policy making, WHO convened diverse stakeholders, including vaccine manufacturers and developers, regulators, and policy makers, across country, regional, and global levels. This report summarises the outcomes of those discussions and proposes potential clinical development scenarios for various target populations, from first-in-human studies to regulatory approval and policy considerations. The intent of this report is to inform the strategies and investment decisions of product developers and funders.

## Status of *Shigella* vaccine development intended for use in LMICs

Both human and animal challenge studies with virulent *Shigella* support the biological feasibility of developing effective *Shigella* vaccines.^7^, ^8^ Additionally, observational studies in endemic areas show that the risk of subsequent disease decreases after *Shigella* infection, consistent with acquired immunity,^9^, ^10^ and indicate a chronological association of protection with age.^9^ Perhaps most compelling are data from efficacy trials with a first-generation O-antigen conjugate vaccine. *Shigella sonnei* O-antigen conjugated to non-toxic recombinant *Pseudomonas aeruginosa* exotoxin A was shown to induce protection in Israeli adults and children older than 3 years.^11^ Of note, the efficacy of this candidate declined with descending age, and the lack of protection was associated with a decrease in serum O-antigen IgG levels. Collectively, these data led to the hypothesis that serum IgG to O-antigen is a correlate of protection for *Shigella* vaccines, and a putative IgG threshold of protection of 1:1600 endpoint titre (Tel Aviv University ELISA) has been proposed for *S sonnei*.^12^ However, significant hurdles for the O-antigen vaccines include serotype-specificity of protective immunity conferred (>50 O-antigen-based serotypes of *Shigella* exist) and immunity required in infants before 12 months of age and the onset of peak incidence.

To overcome the problem of serotype diversity, some vaccine development approaches focus on conserved antigens, such as IpaB, IpaC, IpaD,^13^ and PSSP-1,^14^ in addition to, or instead of, O-antigen. Preclinical challenge studies^8^ and observations from the landmark Global Enteric Multicenter Study^15^ suggest that a multivalent vaccine construct targeting the O-antigen of *Shigella flexneri* 2a, 3a, and 6 (or possibly 1b) and *S sonnei* would provide direct protection against at least 72% of circulating *Shigella* strains and cross-protection for up to 89% of all strains. Thus, several multivalent combinations of next-generation O-antigen-based candidates are under development.^6^, ^16^, ^17^, ^18^ However, it is not yet known whether the immunogenicity of any of these candidates will be significantly higher than the first-generation conjugate *S sonnei* O-antigen recombinant *Pseudomonas aeruginosa* exotoxin A glycoconjugate vaccine in the target population of infants and children in LMICs.

## Potential regulatory approval pathways for *Shigella* vaccines

WHO acknowledges that other target populations (eg, adult travellers in HICs) could enable earlier opportunities for regulatory approval and a more attractive and sustainable market, thereby improving the investment case for *Shigella* vaccine developers. Clarification of earlier regulatory approval pathways for *Shigella* vaccines in different (non-paediatric) target populations could de-risk manufacturer engagement and incentivise funding. Beyond accelerating the path to initial regulatory approval for *Shigella* vaccines in general, WHO seeks to determine and facilitate what is needed to enable uptake and impact in the priority target population of children younger than 5 years in LMICs specifically.

National regulatory authorities have established pathways which outline the evidence required to assess risk versus benefit to support regulatory approval of vaccines for their intended use. Traditional development pathways generally require both safety studies and field efficacy trials, the latter of which can be prohibitive in size, duration, and expense for diseases with lower or unpredictable incidence. For *Shigella*, a phase 3 study based on a clinical endpoint of reduction of acute moderate or severe diarrhoea or dysentery caused by culture-confirmed *Shigella* strains contained within the vaccine would require approximately 7000–15 000 participants, across multiple sites in different countries, over 1–2 years of follow-up.^19^

The COVID-19 pandemic brought about unprecedented speed and new mechanisms for vaccine regulatory approval. *Shigella* vaccine developers are seeking opportunities to leverage these regulatory mechanisms, and (depending on the national regulatory authority or country) potential accelerated approval pathways could include the use of CHIM studies, a route based on a surrogate marker of efficacy, or conditional marketing authorisation (CMA).

*Shigella* CHIM studies are currently performed in naive adults in HICs and provide an efficacy readout. However, this study population does not show the impact of adults living in areas of repeated exposure. To address this, efforts are underway to establish an endemic *Shigella* CHIM at the Kenyan Medical Research Institute/Wellcome Trust Research Programme in Kilifi, Kenya.^20^ The CHIM-based pathway could be considered for supporting the regulatory approval process of *Shigella* vaccines in adult travellers. However, adult CHIM studies conducted in endemic and non-endemic areas will not provide information on whether serum O-antigen IgG is a correlate of protection in infants and children, nor whether the threshold established in adults would translate to this target population.

An accelerated pathway that makes use of a surrogate marker of efficacy would require post-approval effectiveness studies to be completed to confirm the surrogate marker as a correlate of protection, or to show that it provides clinical benefit in real-world conditions, thereby supporting vaccine authorisation for broad use. Cohen and colleagues published evidence in 2022 for a threshold of serum IgG antibodies to *S sonnei* O-antigen as a correlate of protection against shigellosis and reviewed the potential use of this benchmark in facilitating the development of *Shigella* vaccine candidates.^12^

A third accelerated pathway is CMA, in which vaccines could receive regulatory authorisation on less comprehensive clinical efficacy data than typically required, if the benefit of immediate availability outweighs the risk. Additional effectiveness data are then required post authorisation. However, this pathway is typically reserved for use in a public health emergency.

CHIM studies were used in the regulatory authorisation of the Vaxchora cholera vaccine (Emergent Travel Health, Redwood City, CA, USA) for adult travellers living in the USA^21^ and Europe,^22^ and to confirm the surrogate marker of serum vibriocidal antibody seroconversion to support the advancement of this vaccine for its authorisation and use in elderly adults.^23^ CHIM studies also supported the WHO policy recommendation and prequalification of TypBar-TCV typhoid conjugate vaccine (Bharat Biotech International, Hyderabad, India) through demonstration of efficacy and immunobridging to the authorised Vi polysaccharide vaccine.^24^ Immunological bridging of TypBar-TCV to the Typhi Vi polysaccharide vaccine was feasible in children because efficacy of a typhoid conjugate vaccine had previously been shown in Viet Nam.^25^

Establishing and using the O-antigen ELISA or other assays as a correlate and threshold of protection requires harmonisation of the methods for determining antibody titre, and availability of an international reference serum.^26^ To support this effort, collaborative studies coordinated by the UK National Institute of Biological Standards and Controls, involving five centres where *Shigella* ELISAs are routinely performed, and development of a WHO international reference serum for *Shigella* derived from adults immunised with a quadrivalent vaccine, are underway. Consensus documents on standardisation of the primary clinical endpoints and convergence on the clinical study protocol, with alignment on sampling timepoints and optimal immunological assay protocols, have also been published.^7^, ^19^

Three potential regulatory approval pathways that could support different *Shigella* vaccine use cases are described in the following sections, with some common assumptions made. The first assumption is that the indication of use is prevention of moderate or severe diarrhoea or dysentery (or both) caused by *Shigella* strains with the same putative protective antigens that are in the vaccine. Demonstration of vaccine efficacy for regulatory approval based on CHIM studies will be limited to one or two of the circulating serotypes, namely *S flexneri* 2a and *S sonnei.* In a field efficacy study, the primary endpoint could be against a composite of serotypes present in the vaccine. A second assumption is that all pivotal clinical studies submitted in the regulatory data packages described would require safety and efficacy measures, whether through CHIM or field studies, and the use of clinical trial material generated in consistency batches matching the batches to be manufactured at commercial scale. Potential acceleration of clinical study timelines could be offset by the investment and time needed to develop and validate the commercial manufacturing process.

### CHIM-based regulatory approval strategy in adults, for travellers and military personnel

Currently, only two CHIM models exist (*S sonnei* and *S flexneri* 2a) to evaluate proof of concept of a *Shigella* vaccine candidate. O-antigen-based candidates have been evaluated as monovalent constructs, initially in a homologous CHIM, to de-risk the investment in product development of their multivalent combinations. The assumption is that other vaccines incorporating *Shigella* serotype components would also be protective when tested in a homologous CHIM, with the same dose and formulation manufactured by the same process. However, vaccine developers are increasingly moving towards direct proof-of-concept testing of multivalent constructs, to shorten development timelines, and could consider a study using both *S flexneri* 2a and *S sonnei* serotypes simultaneously in a single CHIM study. The CHIMs have been established in the USA at Johns Hopkins University, the University of Maryland, and the Cincinnati Children's Hospital Medical Center.

Assuming that a vaccine intended for use in adults at high risk (eg, travellers and military personnel) in HICs shows efficacy against both the *S flexneri* 2a and *S sonnei* serotypes, there are two potential regulatory authorisation strategies for this indication. First, the vaccine is designed as bivalent from the outset and evaluated in both *S flexneri* 2a and *S sonnei* CHIMs to show homologous protection for each. There is currently one bivalent candidate in development, but it will probably follow a traditional field efficacy study for use beyond HIC adults^27^ and specifically in children. Second, the multivalent candidates for global use are evaluated in both available CHIMs, with the intention of an initial indication for adults in the prevention of disease caused by *S flexneri* 2a and *S sonnei*. This indication could then be expanded if and when similar antibody response titres for the additional (non-CHIM serotypes) are shown, as was the case for the addition of serotypes to pneumococcal conjugate vaccine (PCV7) on the basis of non-inferiority rather than efficacy trials.^28^

**Figure 1:**
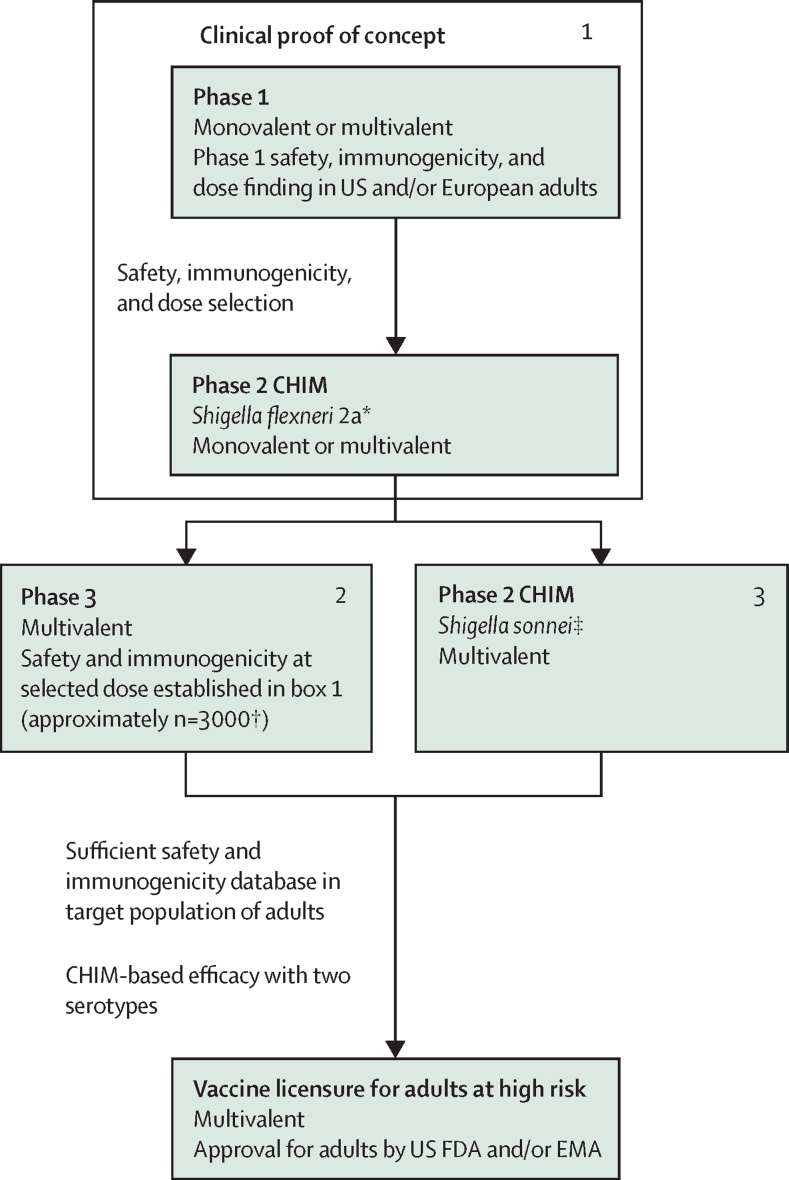
Potential regulatory approval pathway for a *Shigella* vaccine intended for use in adult populations in high-income countries who are exposed to high-risk settings The CHIM studies are interchangeable and could be combined to assess efficacy in the same protocol. CHIM=controlled human infection model. FDA=Food and Drug Administration. EMA=European Medicines Agency. *The study might assess more than one dose. †Minimal safety database. ‡Assume proof of concept against both *S flexneri* 2a and *S sonnei* will be needed.

A phase 1 safety and immunogenicity study in adults can serve as a dose-finding study to select the optimal dose in the phase 2 CHIM study, which is typically assessed in an adult HIC population (figure 1). Because a CHIM study is limited by the number of participants (typically 30–35 per group), a separate phase 3 study in the same population would then be needed to assess the safety and immunogenicity of the selected dose in at least 3000 individuals older than 18 years. The durability of protection could be inferred by assessing the antibody kinetics over time. In parallel, the candidate could be evaluated in a second CHIM with the alternative serotype, to provide an indication of efficacy induced by two constituent antigens.

The assumptions for regulatory approval of a multivalent travellers’ vaccine, intended for use in adults at high risk in HICs, are summarised in the appendix (p 1).

### Traditional efficacy-based regulatory approval route for use in infants and young children in LMICs

**Figure 2:**
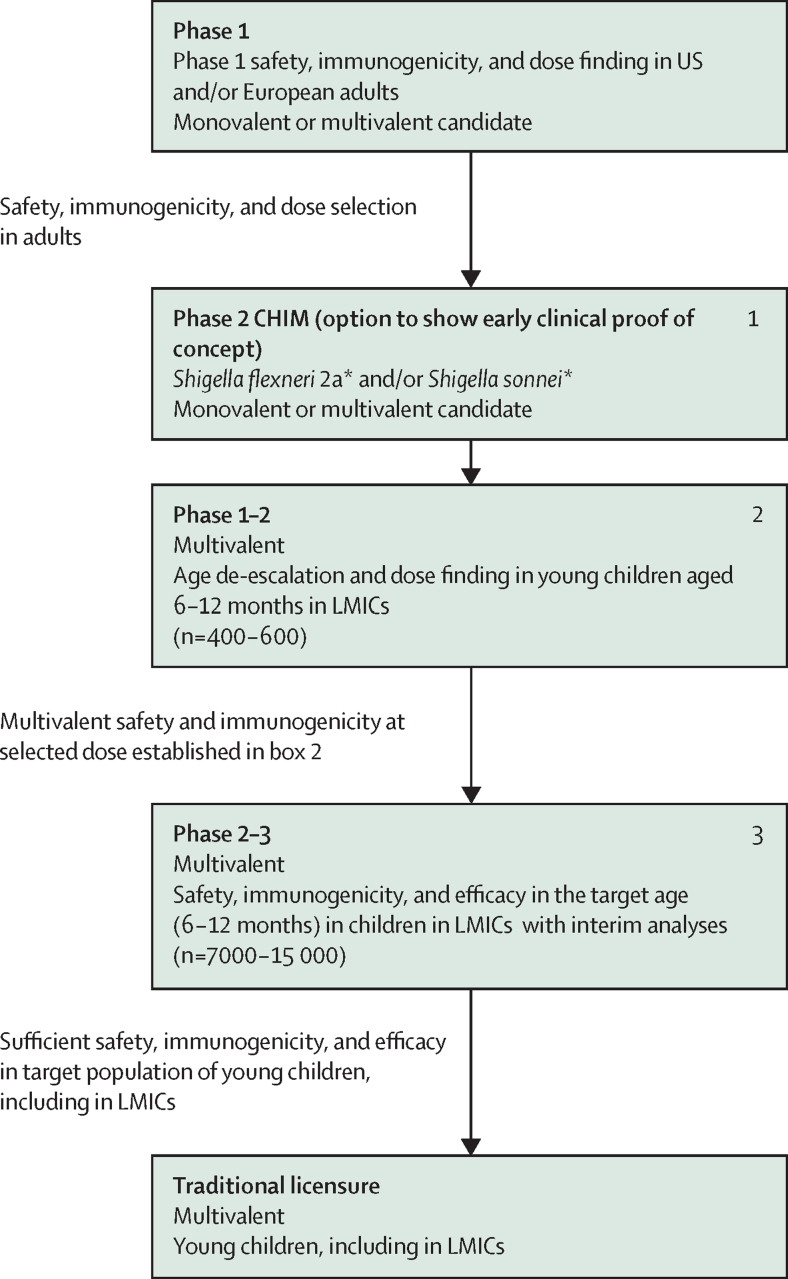
Potential regulatory approval pathway based on a traditional efficacy-based pathway for a *Shigella* vaccine intended to support broad use (based on a global policy recommendation), in infants and children younger than 5 years in LMICs LMIC=low-income or middle-income country. *The study might assess more than one dose.

In the traditional clinical development pathway, the CHIM can be included to establish clinical proof of concept in adults, with the optimal dose of the multivalent candidate in infants or young children in LMICs then defined in a phase 1–2 study (figure 2). A phase 2b–3 study could be in the form of an adaptive trial design that enables prospectively planned modifications (ie, specified in the study protocol and statistical analysis plan) to aspects of the study, based on accumulating data from participants in the trial. It could also include one or more interim analyses to assess data for potential futility once a specified number of cases has been accrued, while the trial is ongoing. The inclusion of an adaptive trial design could allow adjustments for information unavailable at the start of the study and has the potential to ensure optimal statistical efficiency.

National regulatory authorities in endemic countries will require local or translatable clinical data in the form of safety and efficacy in the target population; therefore, the phase 2b–3 study will need to be conducted across multiple countries in the most highly endemic regions to support a global policy recommendation and implementation.

The assumptions for a traditional development and regulatory approval pathway in infants and children younger than 5 years in LMICs are shown in the appendix (p 1).

Mechanisms that facilitate joint review by multiple national regulatory authorities or ethics committees, particularly in the countries intended for use, will help to build consensus on ethical and regulatory questions and common data requirements. One such example is the African Vaccine Regulatory Forum (AVAREF), which works to harmonise regulatory and ethics practices on the African continent in support of product development. Within the framework of the African Medicines Regulatory Harmonization (AMRH) programme, which led to the establishment of the African Medicines Agency (AMA), AVAREF has been selected as the AMRH and AMA technical committee for clinical trials. Therefore, its tools and processes will be used by the AMA and its support provided to AMA for implementation of regulatory reliance. In the context of COVID-19 vaccine development and approval, the International Coalition of Medicines Regulatory Authorities supported strategic coordination and international cooperation among 29 national medicine regulatory authorities, with the WHO as an observer. Finally, the EU-M4all procedure (previously called Article 58) of the European Medicines Agency (EMA) is a mechanism to solicit scientific opinions on high-priority vaccines that are intended for markets outside of the EU, in cooperation with WHO. The process involves local epidemiology and disease expertise of WHO and national regulators in the target countries to provide a single development and assessment pathway that aims to facilitate prequalification of vaccines by WHO and registration in the target countries.^29^

### CMA to expedite the approval time of *Shigella* vaccines for use in infants and young children in non-Gavi countries

Some regulatory authorities, such as the EMA, might be able to grant a CMA based on a less comprehensive clinical data package than typically required under a traditional approach to product approval. However, such expedited regulatory processes are usually limited to vaccines that address a priority public health need, where the level of efficacy observed is exceptional, or where the benefit of immediate availability of the vaccine outweighs the risk inherent in the fact that additional data are still required. If approved at this stage and under such a regulatory process, the data would not be sufficient to warrant WHO policy recommendation or prequalification for broad use. However, the CMA could enable use in some public programmes or private markets, including in LMICs, with the added benefit of generating safety and effectiveness data in the target population, in parallel to completion of the phase 3 efficacy study.

CMA could conceivably be based on an interim analysis of a phase 2b–3 study, potentially supported by O-antigen IgG seroconversion as a read-out. If promising, CMA could be pursued while the phase 3 study continues, as long as the manufacturing process is well characterised and vaccine supply is available. However, this strategy poses a high risk for developers with little certainty of broad use, so the two aforementioned pathways are recommended. The assumptions for CMA in infants and children younger than 5 years, for use in non-Gavi countries (ie, those not eligible for support by Gavi, the Vaccine Alliance), are summarised in the appendix (pp 1–2).

### Fully integrated *Shigella* vaccine regulatory approval pathway

The two aforementioned licensure pathways (in adults at high risk in HICs and in infants and young children in endemic countries) could be pursued in parallel. The pathway to the HIC adult indication is quicker and less costly and could warrant investment in establishing the manufacturing capacity that could also be used to produce paediatric vaccines, once licensed. In addition, the safety and efficacy data derived from use in HIC adults would increase awareness of *Shigella* and its burden and help to generate demand for a paediatric vaccine to be used in LMICs. The benefits and limitations or risks of each pathway are summarised in the appendix (p 2).

## Beyond regulatory approval: evidence to support national and global policy recommendations

The proposed indication for a *Shigella* vaccine in young children is prevention of moderate or severe diarrhoea or dysentery caused by *Shigella* strains with the same putative protective antigens that are in the vaccine.^5^ Once a vaccine has been approved and is available, the National Immunization Technical Advisory Group in each country that intends to use the vaccine will undertake a systematic decision-making process based on a review of the available burden, safety, and effectiveness data, in the context of their specific broader health and economic priorities. Vaccines intended for use in LMICs will benefit from a policy recommendation from the WHO Strategic Advisory Group of Experts on Immunization (SAGE) and WHO prequalification to support introduction decisions at the country level. Both are prerequisites for Gavi financing and UNICEF procurement and are important for decision making in non-Gavi-supported countries. However, WHO policy recommendation and prequalification would only be feasible if the product has full approval for use, and not conditional approval.

Beyond prevention of disease, programmatic fit and evidence of prevention or reduction of *Shigella-*attributable malnutrition and growth stunting, or reduction of antibiotic use, or both, are likely to be important for WHO policy recommendation and financing, as drivers of cost-effectiveness. Early consideration of the criteria and evidence requirements for vaccine recommendation and introduction is needed to avoid any delay in vaccine implementation.

## Current and near-term efforts to support clinical, regulatory, and policy-related strategic planning

Several parallel workstreams are ongoing to prepare for and inform the most expeditious and practical pathway to *Shigella* vaccine approval for use in young children living in LMICs. This work is in anticipation that the pivotal clinical efficacy studies for the leading candidate or candidates will begin in 2025. Efforts are underway to develop and seek input on the design of a multi-country, multi-site phase 3 clinical study for *Shigella* vaccines intended for infants and young children living in endemic countries. These include the development of a data package and evidence to support culture-independent methods to be used for microbiological outcome confirmation against vaccine strains, as opposed to less sensitive culture-based methods, which will considerably reduce sample size requirements to power the robust evaluation of the primary endpoint;^19^ and the establishment of a *Shigella* Vaccine Trial Design Working Group by the Bill & Melinda Gates Foundation with regional representatives, including those from the National Institutes of Health, WHO, and academia, to develop considerations and potential options for a vaccine efficacy trial design, focusing on aspects such as the regulatory acceptability of molecular laboratory confirmation of *Shigella*, clinical case definitions, and trial endpoints.^19^ Additionally, the serum O-antigen IgG ELISA protocol has been standardised and an international reference serum is being developed in collaboration with the National Institute of Biological Standards and Controls and with support from the Gates Foundation and WHO. Surveillance is being conducted through the Enterics For Global Health study to establish serotype-specific incidence and consequences of *Shigella* diarrhoea in children younger than 36 months, within seven country sites in Bangladesh, The Gambia, Kenya, Malawi, Mali, Pakistan, and Peru; these efforts are intended to inform the design, sample size, and implementation of field efficacy trials in the target population and to provide up-to-date data to policy makers on the incidence of shigellosis and antimicrobial resistance burden.^30^ Early scientific advice meetings have been held with regulatory authorities, such as the US Food and Drug Administration and the European Medicines Agency, as well as AVAREF, including a convening of endemic country national regulatory authorities by WHO to discuss clinical trial considerations and regulatory strategies. Other ongoing efforts include: establishment of a WHO working group to evaluate the methodology and data needed to comprehensively and robustly assess the *Shigella* disease burden;^31^ development of a full value of vaccines assessment to articulate the potential demand, use case, and socioeconomic impact of *Shigella* vaccines targeted to infants and young children in LMICs;^32^ and the development of a vaccine value profile to collate existing information on the public health need and potential value of *Shigella* vaccines, which has been commissioned by WHO in preparation for the next cycle of Gavi's Vaccine Investment Strategy (in press).

## Conclusion

*Shigella* infections cause a substantial global public health burden. Although several key target populations would benefit from a vaccine, the priority need resides with children younger than 5 years in LMICs, who experience up to 94 000 deaths annually and serious long-term sequalae that can lead to detrimental socioeconomic consequences.

Evidence for the biological feasibility of developing effective *Shigella* vaccines exists, and efficacy has been shown in adults through targeting antibody responses to the surface O-antigen. Both observational and clinical efficacy studies suggest that O-antigen serum IgG concentrations could be a correlate of protection for O-antigen-containing vaccines, and an immunological threshold of protection has been proposed for *S sonnei*.^12^ If validated in a phase 3 efficacy study in infants and young children, the regulatory approval of next-generation O-antigen-based *Shigella* vaccines that are intended for use in children younger than 5 years, based on immunobridging studies in the target population, could be greatly accelerated. In the meantime, the CHIM accelerates the development of multivalent O-antigen-based *Shigella* vaccine candidates by demonstrating initial proof of concept in adults, providing a potential pathway to regulatory approval of a vaccine for use in adults at high risk living in HICs and in de-risking investment for candidate evaluation in field studies.

Through consultation with regulators, policy makers, and *Shigella* vaccine product developers on three potential pathways, two feasible routes to regulatory approval for next-generation O-antigen-based *Shigella* vaccine candidates were identified: accelerated approval based on a CHIM model or the traditional regulatory approval pathway.

The CHIM model could serve as the basis for accelerated or expedited approval of a vaccine for adults at high risk, while also further facilitating investigation of serum O-antigen IgG responses as a correlate of protection. Although this pathway might be attractive to vaccine developers, the safety and efficacy data for an indication in adults at high risk, based on studies conducted in HICs, will not be particularly relevant to the paediatric LMIC setting. However, the regulatory approval of the vaccine for use in the HIC market could support the development of a sustainable vaccine manufacturing base and vaccine availability for LMICs with this dual-market vaccine.

The most plausible pathway for *Shigella* vaccine approval and broad uptake in children younger than 5 years in LMICs is through the traditional regulatory approval pathway, based on a field efficacy study across multiple trial sites with comparatively high *Shigella* incidence. This pathway enables the generation of a robust safety database, evidence of vaccine impact across primary as well as secondary and exploratory endpoints (which could include morbidity, linear growth faltering, and parameters such as antimicrobial resistance). Moreover, it offers the opportunity to investigate the impact on the constituent serotypes for which CHIMs are not available, acknowledging that it remains uncertain to what extent efficacy across serotypes can be gathered in a pre-approval field efficacy study. Although protection against non-CHIM-evaluated serotypes is expected to be similar to that against serotypes included in the current *Shigella* CHIM, there is a risk that protection will differ. Perhaps most importantly, the evidence generated through a traditional efficacy study is currently the only pathway that will be able to inform a SAGE policy recommendation to WHO, which considers aspects such as vaccine programmatic fit, cost-effectiveness, and acceptability.

Within the traditional regulatory approval pathway, there is the theoretical possibility of CMA, based on interim efficacy analysis. However, as discussed herein, this pathway is unlikely outside of the context of a public health emergency and presents the most high-risk strategy for developers, regulators, and policy makers.

A WHO policy recommendation is a prerequisite for WHO prequalification, and for financing by Gavi. Should a product become available, it is expected that Gavi would evaluate it through its Vaccine Investment Strategy, which is conducted on a 5-year cycle. The Vaccine Investment Strategy is centred around a robust and transparent mechanism for evaluation of vaccine products based on a number of criteria, which include health and economic impact, contribution of equity and social protection, feasibility, and implementation costs. Even as countries graduate from Gavi eligibility, they are likely to consider a WHO policy recommendation to be crucial for government decision making to introduce a new enteric vaccine into the national immunisation programme. For this reason, generation of the data and evidence needed for both policy making (whether at the national or global level) and Gavi financing can accelerate the pathway to vaccine introduction and use in countries and target populations with the greatest need.

To accelerate the global availability of a *Shigella* vaccine, the pathways described here should advance in parallel. The historical epidemiology of *Shigella* disease and current incidence, considering demographic and climatic factors, portend a real possibility of major disease outbreaks and continued high levels of morbidity and mortality, for which the accelerated introduction of safe and effective vaccines could have major impact. Finally, it is important to emphasise that vaccine development for use in adults at high risk in high-resource settings should not delay vaccine development for use in young children in low-resource, endemic country populations, where the need is greatest.

## Declaration of interests

NB provides regulatory advice to the regulated industry. PBP receives funding from the Bill & Melinda Gates Foundation for Enterics for Global Health Shigella surveillance. DCK received funding from the Bill & Melinda Gates Foundation while at PATH, and chaired the WHO Product Development for Vaccines Advisory Committee until 2022. MSR consulted for Emergent Biosolutions and Limmatech Biologics while at the University of Nevada. All other authors declare no competing interests.
