# Supplementary material for: Clinical and regulatory development strategies for Shigella vaccines intended for children younger than 5 years in low-income and middle-income countries
Source: Lancet Glob Health. 2023 Oct 17;11(11):e1819–26. doi: 10.1016/S2214-109X(23)00421-7 (PMC10603611; doi:10.1016/S2214-109X(23)00421-7)
Supplement: Supplementary appendix [file mmc1.pdf]

THE LANCET
Global Health

Supplementary appendix

This appendix formed part of the original submission and has been peer reviewed. We post it as supplied by the authors.

Supplement to: Giersing BK, Isbrucker R, Kaslow DC, et al. Clinical and regulatory development strategies for *Shigella* vaccines intended for children younger than 5 years in low-income and middle-income countries. *Lancet Glob Health* 2023; **11:** e1819–26.

Clinical and regulatory development strategies for *Shigella* vaccines intended for uptake and impact in infants and young children, living in low- and middle-income countries

Appendix

Box 1: Summary assumptions and inter-dependencies for regulatory approval of a multivalent travellers' vaccine, intended for use in high-risk adults in HICs:
▪ The methodology is harmonised across the *S. flexneri* 2a and *S. sonnei* CHIMs, the O-Ag IgG ELISA assays for all vaccine serotypes are standardised, and an international reference serum against all vaccine serotypes is established;<br>▪ The CHIM studies consider a 3- or 6-month efficacy assessment time point based on precedence of the Vaxchora vaccine approval approach [38, 39].<br>▪ Co-administration studies with other common travel vaccines may need to be conducted to support non-interference, pre- or post-licensure.<br>▪ The development of CHIM for the additional *Shigella flexneri* serotypes (3a and 6) may serve to further de-risk investment in product development of a multivalent vaccine; however, demonstration of efficacy for 2 serotypes in a CHIM is likely considered sufficient to support licensure of the multivalent vaccine for high-risk adults in HICs, assuming similar immune responses against the other serotypes are demonstrated.

Box 2: Summary assumptions and dependencies for a traditional development and regulatory approval pathway in infants and young children under 5 years of age in LMIC countries
▪ A multi-country, multi-region epidemiology study is needed to establish the site-specific incidence of moderate or severe diarrhoea or dysentery caused by *Shigella*, in children under the age of 36 months, as well as to assess serotype prevalence. The incidence and serotype data are foundational to clinical trial design, including the sample size, and the *Shigella* force of infection. These studies are ongoing [53].<br>▪ The efficacy will likely only be measurable against a composite endpoint of all *Shigella* vaccine serotypes, or at most, 1 or 2 *Shigella* serotypes (likely *S. flexneri* 2a and/or *S. sonnei*); however, serum IgG against the O-Ag could be explored as an immune marker to infer protection from the other strains. If this study validates a serum IgG threshold against O-Ag for *S. sonnei* or *S. flexneri* 2a as a correlate of protection, future O-Ag-based *Shigella* candidates could be licensed on the basis of safety and non-inferiority in an immunogenicity study in the target population.<br>▪ Compatibility of the vaccine within the schedule and delivery setting of the expanded programme of immunization (EPI) will be crucial for cost-effectiveness, which is a pre-requisite for global policy recommendation and country-level introduction.

Box 3: Summary assumptions and dependencies for conditional marketing authorisation in infants and young children under 5 years of age, for use in non-Gavi countries:
▪ The public health need for a *Shigella* vaccine is considered an emergency, and the interim data are sufficiently compelling to qualify for the CMA pathway.

▪ The phase IIb component of the study has sufficient subjects to support a safety database of at least n=3,000.<br>▪ The phase IIb/phase III study is a multi-country study in areas of high *Shigella* incidence; this would require regional regulatory co-ordination and oversight.<br>▪ Efficacy data from CHIM would be supportive in CMA scenario, although CHIM data would be from adults.

Table 1: Summary of potential Shigella vaccine licensure strategies, and their relative benefits, limitations, and risks.

Regulatory approval strategy	Benefits	Limitations/Risks
CHIM-based pathway in high-risk adults in HICs	▪ Addresses the need in some key populations<br>▪ Shorter and less costly development pathway may be an incentive for developers<br>▪ Builds effectiveness, acceptance and pharmacovigilance database that will support paediatric licensure<br>▪ May accelerate the timeline to approval and access for children in LMICs as manufacturing process would be established<br>▪ De-risks subsequent expansion of the indication to under 5s in LMICs<br>▪ May help to sustain vaccine availability through multiple manufacturers	▪ Based on precedence of Vaxchora, initial approval may be with limited duration of protection (3-6 mo) depending on clinical trial design;<br>▪ The critical product attributes for a HIC high-risk adult vaccine differ from those for under 5s in LMICs, i.e. duration of protection, schedule, number of doses, presentation. Vaccine must be designed for use in children in LMICs from the outset if broad indication is envisaged.<br>▪ May detract funding/resources from indications with a greater public health need, i.e. children in LMICs.
Traditional efficacy-based pathway in infants and young children in LMICs	▪ Most direct route to generating data and evidence in the priority target population<br>▪ Most risk averse strategy from the perspective of regulators and policy makers<br>▪ Provides opportunity to generate evidence on implementation feasibility and build demand, as part of, or in parallel to phase 3 study, to accelerate policy and introduction decisions following licensure.<br>▪ Pathway most likely to accelerate and ensure equitable access to young children in LMICs through a WHO policy recommendation, and regional and national level policy decision makers.	▪ Longest, and most costly route to licensure<br>▪ Requirement for funding and partnership with large vaccine manufacturers potentially introduces bottlenecks, particularly if the manufacturer prioritizes the travellers' indication.

Conditional marketing authorization in under 5’s	▪ May enable earlier use of a priority vaccine in the target population of young children, while efficacy study ongoing	▪ Limited to vaccines where there is a compelling public health need, in situations where interim efficacy is exceptional, and where the anticipated clinical benefits far outweigh the potential risks.<br>▪ Regulators will be unlikely to approve through CMA without commitment to the continuation of the efficacy study, per protocol.<br>▪ High-risk strategy for developers.<br>▪ Will limit initial availability to the private market, and lead to inequitable access
